# A randomized trial of a wearable UV dosimeter for skin cancer prevention

**DOI:** 10.3389/fmed.2024.1259050

**Published:** 2024-03-01

**Authors:** Emmanuel L. P. Dumont, Peter D. Kaplan, Catherine Do, Shayak Banerjee, Melissa Barrer, Khaled Ezzedine, Jonathan H. Zippin, George I. Varghese

**Affiliations:** ^1^Shade, Nutley, NJ, United States; ^2^Hackensack Meridian Center for Discovery and Innovation, Nutley, NJ, United States; ^3^Department of Pathology, New York University Langone Health, New York, NY, United States; ^4^Department of Dermatology, Weill Cornell Medicine, New York, NY, United States; ^5^Department of Dermatology, University Hospital Henri Mondor, Créteil, France

**Keywords:** skin cancer, basal cell carcinoma, squamous cell carcinoma, photoprotection, ultraviolet exposure

## Abstract

**Background:**

Non-melanoma skin cancer (NMSC) is the most prevalent cancer in the United States. Despite guidelines on ultraviolet (UV) avoidance, it remains difficult for people to assess their exposure, as UV is invisible and the onset of UV-induced symptoms is delayed.

**Methods:**

In a prospective randomized trial, 97 elderly patients with a history of actinic keratoses (AK) were followed over 6 months. Fifty patients received UV counseling from a dermatologist and a wearable UV dosimeter that provided real-time and cumulative UV exposure. Forty-seven patients received only UV counseling from a dermatologist.

**Results:**

Over 75% of participants recorded UV exposure at least once a week during the summer. After 6 months of intervention, when comparing the device group to the control group, we observed a non-significant 20% lower ratio of incidence rates of AKs (95% CI = [−41, 55%], *p*-value = 0.44) and a significant 95% lower ratio of incidence rates of NMSCs (95% CI = [33, 99.6%], *p*-value = 0.024). Surveys demonstrated that the control group’s score in self-perceived ability to participate in social activities significantly increased by 1.2 (*p*-value = 0.04), while in the device group, this score non-significantly decreased by 0.9 (*p*-value = 0.1). We did not observe changes, or between-group differences, in anxiety and depression surveys.

**Conclusion:**

This pilot clinical trial has a short duration and a small sample size. However, device adherence and quality of life questionnaires suggest a smartphone-connected wearable UV dosimeter is well accepted by an elderly population. This trial also indicates that a wearable UV dosimeter may be an effective behavioral change tool to reduce NMSC incidence in an elderly population with a prior history of AKs.

**Clinical trial registration**: clinicaltrials.gov, identifier NCT03315286.

## Background

Skin cancer is the most common cancer in the United States, affecting more than 3 million Americans per year (1), and its incidence is still on the rise worldwide (2). Genetic, phenotypic, and environmental factors, specifically ultraviolet (UV) radiation, are considered the largest contributing factors to the development of skin cancer (3). Over the past three decades, there has been a push towards protecting the skin from the dangers of UV through educational campaigns about the harmful effects of UV (4), topical application of physical and chemical blockers in sunscreens (5), UV protective clothing (6), and vitamin supplementation such as niacinamide (7). More recent controversies on the effectiveness (8) and safety (9) of sunscreens have created a critical need for safer strategies to help reduce the overall UV exposure to our skin. Public health agencies, like the EPA, have published guidelines using the forecasted UV index (UVI) per zip code. This information is easily accessible but it provides an estimate of the sun’s strength in one’s zipcode, so it does not take into account one’s location-driven UVI variations (e.g., cloud cover, shade of a building) and one’s duration of exposure to UV. On the other hand, the International Commission on Non-Ionizing Radiation Protection recommends a daily cumulative UV exposure limit of 30 erythemaly-weighted Joules per square meter (equivalent to 30% of one Standard Erythema Dose (10–13), or “SED”) for direct exposure to eyes or the skin (14). However, this limit of cumulative UV exposure cannot be easily measured without a wearable UV dosimeter. Among this new class of wearables, the Shade UV sensor accurately records erythemaly-weighted UV exposure and has reached standard benchmarks making it superior to other wearable dosimeters (15, 16). However, wearable UV dosimeters possess inherent limitations, as their measurements may not accurately reflect the UV exposure of various sun-exposed body parts or capture the variations in cumulative exposure across different locations. In this prospective, randomized clinical trial, we assessed the clinical efficacy of the Shade UV sensor and its companion mobile application on pre- and cancerous lesions against the standard of care over six months overlapping a summer in an elderly patient population disposed to developing skin cancer.

## Methods

### Study design

This prospective, randomized, observer-blinded, controlled clinical trial enrolled elderly patients with a history of actinic keratoses at a single site in New York City, NY. The trial was conducted under the oversight of the Institutional Review Board (IRB) of Weill Cornell Medicine and the National Cancer Institute (NCI). It adhered to applicable governmental regulations. The IRB and the NCI approved the protocol and the consent forms. As a requirement of contract HHSN261201700005c with NCI, the protocol and all amendments were submitted and approved by the program officer. All participants provided written informed consent before enrollment. The sponsor, YouV Labs, Inc., and the trial’s principal investigator (GV) were responsible for the overall trial design, site selection, monitoring, and data analysis. Investigators were responsible for data collection, recruitment, and treatment. The authors vouch for the accuracy and completeness of the data and the fidelity of the trial to the protocol. The trial was registered on clinicaltrials.gov under the identification NCT03315286 on October 20, 2017.

### Participants, randomization, and data blinding

Eligible participants were individuals aged 18 years or older who had a history of actinic keratosis (AKs), with at least one AK diagnosed clinically in the 12 months before enrollment, or a minimum of five clinically diagnosed AKs in the 5 years before enrollment. Patients having received UV therapy in the past 6 months or field therapy for the treatment of actinic keratosis in the past 3 months were excluded. Participants were assigned using randomly-generated blocks of four, stratified by skin type, to receive a wearable UV dosimeter and standard-of-care UV education or solely standard-of-care UV education (avoid going outside between 10 am and 4 pm, apply sunscreen with SPF 30–50 and re-apply every 2 hours including when coming out of the water, wear sun protective clothing, such as a hat). The UV education was provided in person by the study dermatologist at the end of all three clinical visits. Patient adherence to these guidelines was not measured as primary endpoints in our clinical trial. We used randomization in blocks of four to balance seasonal trends in UV exposure. All participants received $50 per visit (up to $150 across the study) to cover for their visit co-pays and transportation, and participants receiving a dosimeter were encouraged to wear it every day and received a compliance payment of $20 per visit if their dosimeter recorded UV at least 2 days per week (up to $40 across the study). The compliance payments were designed to encourage participants to wear the dosimeter at least during the weekend. Participants were enrolled from April to July 2018 and had two follow-up visits at 3 months intervals. The final visits ran from November to January 2019. All participants from both groups were examined by the same dermatologist who was blinded to their group assignment.

### Wearable UV dosimeters

**Figure 1:**
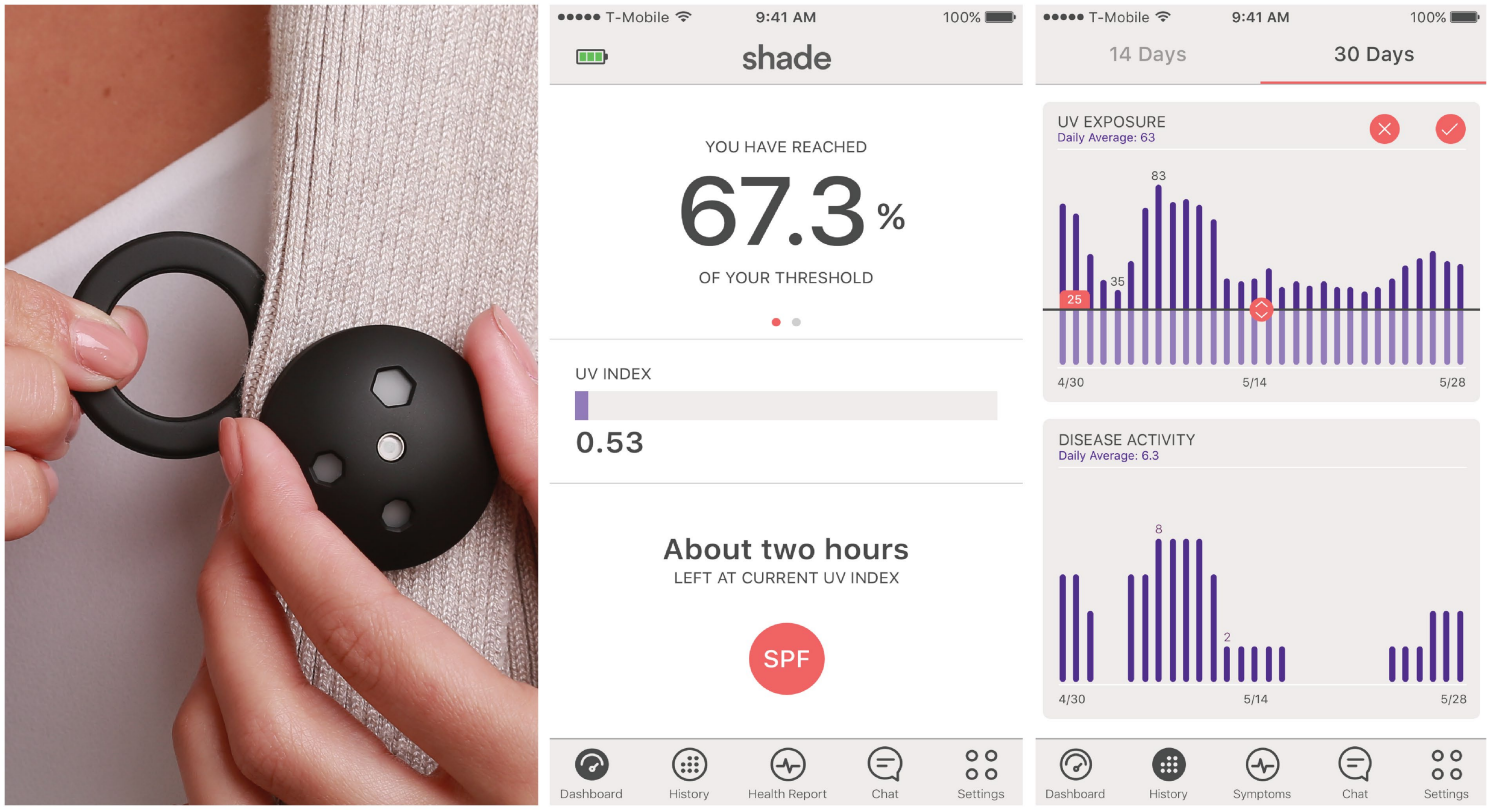
Wearable ultraviolet dosimeter, its magnet, and its companion smartphone application. The erythemaly-weighted UV measurements by the dosimeter were referred to as “UV index” for simplicity in the mobile application.

The sponsor provided the Shade UV dosimeters (16) and a companion smartphone application. The dosimeters measured the erythemaly-weighted UV exposure every second and aggregated the cumulative dose every 6 min. They were designed to be worn on the chest using a magnetic attachment (Figure 1). The sponsor developed an application for both Apple and Android smartphones connected to the UV dosimeter via Bluetooth. The smartphone application displayed a real-time UV index, real-time cumulative UV exposure, and historical data of daily UV exposure. At enrollment, the device participants were trained to use the dosimeter and select a daily UV dose threshold on the application. This threshold was customizable through the application and could be changed by the participant. Participants’ daily UV exposure would reset to zero at midnight and, as it increases throughout the day, would be compared to the threshold they had set. With every further attainment of 20% of the daily UV dose threshold, the app pushes a smartphone notification (e.g., “You have reached [20%, 40%, 60%] of your daily UV dose”). Participants could also inform the smartphone app if they were using sunscreen by indicating the overall SPF but not the body location of the application. The cumulative UV exposure would be divided by the sun protection factor (SPF) during the two hours following sunscreen application before being added to the daily UV exposure (17).

### Safety assessments

Safety assessments included monitoring of adverse events related or possibly related to the device or sun exposure experienced within the study period.

### Efficacy assessments

A single, blinded dermatologist counted AKs and NMSCs on sun-exposed areas (scalp, face, hands) at enrollment and at each subsequent visit (3 months after enrollment and 6 months after enrollment). Pictures of every lesion and its locations were recorded. AKs may manifest clinically as keratotic macule(s) or papule(s) on an erythematous base. To ensure that only new AKs or NMSCs after enrollment were counted, each lesion’s location and picture were compared to prior lesions (AK or NMSC). The primary endpoint was the incidence rate of AKs at disenrollment compared to the intermediary visit. Secondary clinical endpoints included the incidence rate of NMSC at disenrollment compared to the intermediary visit. All AKs were treated and eliminated at the time of each visit with cryotherapy, ensuring an accurate calculation of the longitudinal AK incidence. All lesions suspected of being cancerous were biopsied, and a blinded pathologist confirmed the diagnoses. The dermatologist would surgically remove a cancerous lesion if a patient were diagnosed with it. Other secondary endpoints included scores on three NIH PROMIS 8-question surveys on anxiety, depression, and the ability to participate in social activities.

### Data entry

Case Report Forms (CRFs) were filled out by participants, the study coordinator, and the dermatologist on paper. CRFs were monitored by the sponsor for completeness, consistency, and agreement with underlying medical records periodically during the study. During monitoring, the sponsor, however, did not know if a CRF belonged to an intervention or a control participant, minimizing the risk of influencing the outcome of the trial if it were to modify the CRFs.

### Statistical analysis

We first compared all collected clinico-demographic features to identify imbalances between the control and intervention groups. All feature showing a difference between groups (*p*-value <0.2) was selected for the subsequent multivariate analyses, regardless of their potential association with AK or cancer (Table 1). These features were age (*p* = 0.0001) and gender (*p* = 0.119).

**Table 1:** Demographic and clinical characteristics.

Characteristics	Control (*N* = 43)	Device (*N* = 49)	Total (*N* = 92)	*p*-value
Gender – no. of participants (%)				0.119
Male	24 (56%)	35 (71%)	59 (64%)	
Female	19 (44%)	14 (29%)	33 (36%)	
Mean age (SD) – yr	69 (7.0)	64 (10)	66 (9)	0.0001 (*)
Race – no. of participants (%)				n/a
White	43 (100%)	49 (100%)	92 (100%)	
Non White	0 (0%)	0 (0%)	0 (0%)	
Ethnicity – no. of participants (%)				0.494
Hispanic or Latino	0 (0%)	2 (4%)	2 (2%)	
Not Hispanic or Latino	38 (88%)	39 (80%)	77 (84%)	
Unknown	5 (12%)	8 (16%)	13 (14%)	
Fitzpatrick type – no. of participants (%)				0.429
Type 1	11 (26%)	19 (39%)	30 (33%)	
Type 2	27 (63%)	26 (53%)	53 (58%)	
Type 3	5 (12%)	4 (8%)	9 (8%)	
Education – no. of participants (%)				0.931
Did not complete college	5 (12%)	6 (12%)	11 (12%)	
Completed college	37 (86%)	42 (86%)	79 (86%)	
Unknown	1 (2%)	1 (2%)	2 (2%)	
Risk factor for skin cancer – no. of participants (%)				
Being diagnosed with a cancer at enrollment	4 (9%)	8 (16%)	12 (13%)	0.49
Current smoker	1 (2%)	1 (2%)	2 (2%)	1.000
Regular user of a tanning bed	1 (2%)	0 (0%)	1 (1%)	0.467

SD, standard deviation; no., number. All demographic characteristics were reported by the participant except for the Fitzpatrick skin type, reported by the clinical principal investigator. For categorical covariates, *p*-values were calculated using Chi-square tests (gender, education, and being diagnosed with skin cancer at enrollment) and Fisher’s exact tests when the Chi-squared test requirement was not met (ethnicity, smoking status, skin type, use of a tanning bed). We used a Spearman *t*-test for the age.

Incidence rates (IR) of AK and NMSC within 3 months intervals at the intermediary visit and disenrollment were calculated using a multivariate Poisson model controlled for age and gender. The incidence rate ratio (IRR) between groups was calculated using a longitudinal approach, comparing the changes in IRs between the intermediary visit and disenrollment in each group, and controlled for age and gender. The trial was designed for the null hypothesis that the efficacy of the UV dosimeter is less than 25% in reducing the rate of newly-formed AKs over 3 months in a population of 102 participants. Analyzing over 7,000 patient visits from January 31, 2013 to January 31, 2018 at the department of dermatology at Weill Cornell Medicine, we applied Monte Carlo simulations to determine that a 25% decrease in the number of AKs would be significantly observed (Student’s *t*-test, *p* < 0.05; power ≥80%) across a population of 102 participants.

## Results

### Trial population

**Figure 2:**
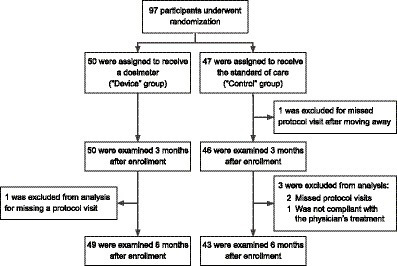
Randomization and analysis populations.

Between April 1, 2018, and July 31, 2018, 97 patients underwent randomization. 50 were assigned to the device group and received a Shade UV sensor and UV protection counseling. 47 were assigned to the control group and received UV protection counseling only (Figure 2). Skin type, defined by the Fitzpatrick scale (from 1 to 6) (18), was balanced between the device and the control group (Table 1). Gender, skin type, ethnicity, race, education, and known skin cancer risk factors were balanced in the two groups. The mean age of the participants was 66 years. Despite randomization, the participants in the device group were significantly younger than the participants in the control group by 5 years on average.

### Safety

No adverse events were reported during the trial.

### Efficacy

**Figure 3:**
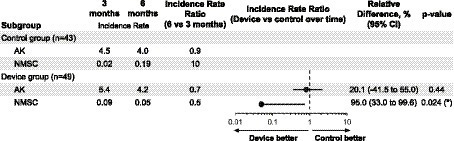
The incidence rate of new actinic keratosis (AK) and non-melanoma skin cancer (NMSC) at 3 and 6 months in the intervention group. The incidence rate ratio (the ratio of the changes in incidence rates in the two groups, IRR), relative differences (1 − IRR, multiplied by 100), and *p*-values are estimated from a Poisson model that includes all variables whose *p*-value is below 0.2 as covariates (gender and age). When the covariates gender and age were omitted from the model, the conclusions remained unchanged. The ratio of incidence rates of NMSCs at 6 months was significantly lower in the device group than in the control group (relative difference: 95.0%, *p*-value = 0.024). This benefit with the device was also observed for AKs but not significantly (relative difference: 20.1%, *p*-value = 0.44). In the supplementary information, a detailed analysis of Basal Cell Carcinoma (BCC) and Squamous Cell Carcinoma (SCC) is included. Given the higher prevalence of BCC compared to SCC, the findings might predominantly apply to BCC cases.

In Figure 3, we present the incidence rates for AK and NMSC at the intermediary visit (3 months after enrollment) and disenrollment (after summer, 6 months after enrollment). Six months into the intervention, when comparing the device group to the control group, we measured a non-significant 20% lower ratio of IRs of AKs (95% CI = [−41, 55%], *p*-value = 0.44) and a significant 95% lower ratio of incidence rates of NMSCs (95% CI = [33, 99.6%], *p*-value = 0.024).

Each PROMIS form has 8 questions rated from 1 to 5, for a combined score between 8 and 40. We found a significant relative decrease of 2.1 points (*p*-value = 0.010, 95% CI: −3.69, −0.50) in self-reported ability to participate in social events in the device group compared to the control group. We did not measure any difference in anxiety or depression.

### UV behavior

**Figure 4:**
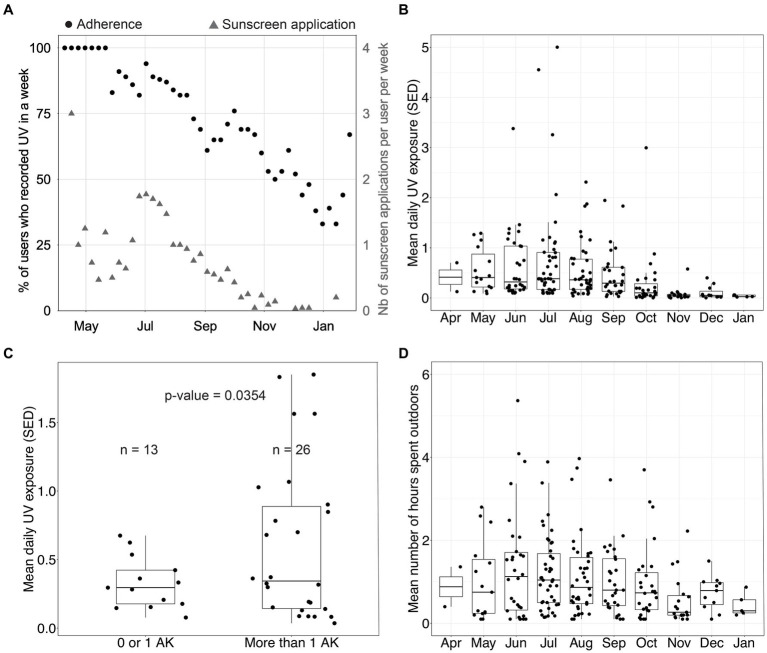
UV exposure data in the device group. **(A)** Percentage of participants who recorded ultraviolet (UV) exposure in a week and number of sunscreen applications per user per week. **(B)** Distribution of the mean daily UV exposure per month (one data point per participant per month). **(C)** Distribution of the mean daily UV exposure over August and September as a function of the number of actinic keratoses (AKs) measured at disenrollment among participants. Using Welch’s 2-sample *t*-test, we found that the group with a low number of AKs had a mean UV exposure of 0.33 standard erythema dose (SED), and the group with a high number of AKs had a mean UV exposure of 0.60 SED (*p*-value = 0.0354). **(D)** Distribution of the mean daily time spent outdoors per month (one data point per participant per month).

Figure 4A displays weekly device compliance and sunscreen use over time. As explained above, sunscreen-related data recorded in this study were self-reported. Therefore, we only report them as descriptive data. Weekly device compliance is approximated by registering UV once a week. Sunscreen usage was measured by the number of self-reported sunscreen applications through the mobile application. The device compliance remained above 75% for most of the summer and dropped below 50% after November, which is unsurprising given the low levels of UV in New York at that time. On average, participants reported applying sunscreen once or twice per week over the summer, a frequency markedly lower than dermatological recommendations. This deficiency in proper sunscreen application is likely to escalate the risk of developing skin cancer.

Figures 4B,D show the mean daily UV exposure and the mean number of hours spent outdoors per month per participant. These were approximated by using the number of hours the sensor was recording UV exposure. Figure 4C clusters participants by the number of AKs diagnosed at disenrollment into two groups and displays the average daily UV exposure over August and September. Device group participants with more than one AK at disenrollment experienced a daily average of 0.60 SED across August and September. In contrast, participants with 0 or 1 lesion experienced a daily average of 0.33 SED across August and September (*p* = 0.0354, Welch’s *t*-test). This data suggests that sub-erythemal chronic exposure beyond 0.34 SED, as measured on the trunk, may contribute to the appearance of lesions on sun-exposed skin. This observation gains additional significance when considering that the International Commission on Non-Ionizing Radiation Protection posits a daily exposure threshold of 0.3 SED to mitigate the enduring impact of UV radiation on the skin and eyes (14). Our analysis, however, does not control for potential confounding factors as this was not the primary endpoint studied. Therefore, additional evaluation is needed.

## Discussion

This randomized clinical trial was designed to evaluate a novel sun protection strategy over 6 months overlapping one summer where real-time, accurate UV information with personalized alerts is provided to participants against the standard of care in UV education. It was powered to detect a 25% reduction in the incidence rate ratio of newly-formed AKs. Our trial was underpowered (92 completed the study vs. 102 participants to reach power), which explains why the 20% lower ratio of IRs of AKs in the device group compared to the control group is not significant. However, Figure 4C shows that, in the device group, participants with more than two AKs at disenrollment had a significantly higher average daily UV exposure than participants with less than one AK diagnosed at disenrollment (*p* = 0.035). The non-significant decrease in the incidence of AK in the device group compared to the control group suggests that real-time measurement of UV exposure using a wearable UV dosimeter could help patients manage their UV exposure and complement standard prevention recommendations.

Additionally, we measured a statistically significant 95% lower ratio of incidence rates of NMSCs (*p*-value = 0.024, 95% CI: [33, 99.6%]) after controlling for all variables whose *p*-value was below 0.2 (age and gender). While these findings suggest that managing UV exposure using real-time and personalized UV information might be useful to prevent UV-related NMSCs, the strong reduction of NMSC incidence rate over only six months is surprising at first. Although we cannot rule out that the observed incidence rate ratio of NMSCs might occur by chance through random sampling, it is unlikely as the *p*-value of 0.024 indicates that there is only one chance in forty that our observation is a false positive. Also, given the low number of NMSC measured during the trial, the 95% confidence interval of the incidence rate ratio ranges from 33 to 99%, so we believe a larger trial would show an impact of the dosimeter on the NMSC incidence rate ratio closer to the effect size measured for AKs (20%). Finally, our observations are consistent with the current cancer biology and epidemiology knowledge which we detail below.

From an epidemiology standpoint, carcinogenesis arises from the accumulation of driver mutations over years or decades (19). However, the risk of NMSC, like several other cancer (20), increases exponentially with age (21). Therefore, as people age, the amount of cumulative UV exposure required to induce NMSC reduces exponentially to the point where only a few weeks of summer UV exposure may be necessary to induce NMSC. This exponentially increasing risk of cancer has been well explained by the percolation theory, which models human tissue as a network of elements whose probability of transitioning from non-cancer to cancer follows a sudden and dramatic increase as driver mutations accumulate (22). For these reasons, a drastic reduction in exposure to one of the most important driver mutations for skin cancer, UV exposure, could lead to an improvement in NMSC incidence over a few months when UV is at its highest (summer). Notably, our trial is not the first to measure an intervention’s impact on skin carcinogenesis over a short period. In 2015, Chen et al. demonstrated through a randomized clinical trial that nicotinamide significantly reduces the incidence rate of NMSC over just 12 months in an elderly population (7). Finally, the two NMSCs reported at disenrollment in the intervention group occurred in participants who had ceased using the device just days after enrollment. This provides additional evidence of the impact of the wearable UV dosimeter on NMSC incidence.

From a molecular biology standpoint, our result is consistent with two established molecular mechanisms. The first one is related to p53 immunopositive epidermal keratinocytes, also called p53 “patches.” These p53 patches follow UV exposure (23) and are associated with skin carcinoma, with 50% of all skin cancers expressing these mutations (24, 25). The prevalence of p53 patches increases with age until saturation when people reach the age of 60 years old (26). Using a murine model, Rebel et al. showed that squamous cell carcinomas (SCC) start appearing after p53 patch saturation, and that the SCC count grows exponentially with time when mice continue to be exposed to daily UV (27). Together, these data indicate that the percolation critical transition for skin cancer would occur when the skin is saturated with p53 patches. Once saturated with p53 patches, additional UV-induced driver mutations are exponentially more likely to lead to skin cancer. In addition, UV radiation induces immunosuppression, which in turn triggers a rapid development of NMSC (28). UV radiation can induce immunosuppression via various mechanisms, including direct immune cell activation and the activation of suppressor immune cells (29). Both UVA and UVB have distinct effects on immune cell function, and it is possible that seasonal reduction in UV exposure in our device group may have allowed for increased immune surveillance and NMSC clearance. Together, these biological mechanisms provide a possible rationale for the deceleration of NMSC development in an elderly population following UV avoidance, even after a few months.

Finally, we found a significant relative decrease of 2.1 points (*p*-value = 0.010, 95% CI: −3.69, −0.50) in self-reported ability to participate in social events in the device group compared to the control group. We did not measure any difference in anxiety or depression. This result suggests that real-time UV data and feedback increase participants’ awareness of UV, while UV counseling leads participants to become overconfident.

There are several limitations to this study. First, all patients were at high risk of skin cancer, as they had been previously diagnosed with AKs, making them perhaps more sensitive to an intervention. Second, the number of NMSCs is low, so we could not perform stratified analyses by squamous cell carcinoma and basal cell carcinoma, as such analyses would have been underpowered. The breakdown of NMSC by type and body location is available in the supplementary information. Given the higher prevalence of BCC compared to SCC, our findings might predominantly apply to BCC cases. Also, the study population came from a single recruiting site in New York City, where the highest UVI ranges from 6 and 9 during the summer. It is likely that the impact of the device would vary depending on the UV of the recruiting sites, with a lower impact in low UV versus high UV regions. While this hypothesis needs to be confirmed in a multicentric study, our study provides a baseline estimate of the efficiency of a wearable dosimeter as a preventive tool in regions with similar UV exposure. In addition, over 85% of our participants completed college, twice the national average; although this could limit the generalization of our findings to the US population, we did not observe any significant impact of education on the impact of the device as the unadjusted incidence rates of NMSC at disenrollment in the device and control groups stratified by education. Besides, the population was followed for one summer only, leaving the possibility that the device’s impact would be short-lived. UV dosimeters, like sunscreen, are seasonal tools mostly used when UV is at the highest, as shown in Figure 4A. Another limitation of our trial is that we do not know the UV exposure behavior in the control group, so we cannot establish that the device participants have lower UV exposure than the control participants. We chose not to survey control participants’ UV exposure based on their recollection because these surveys are unreliable (30, 31). We also wanted a clean comparison to standard-of-care, and we were concerned that simply wearing the device could influence patients’ UV behavior in the control group (32). Further, wearable UV dosimeters used in this trial measure UV exposure from a single location (the trunk), which is not necessarily representative of sun-exposed body locations (e.g., hands, face, or scalp). Selecting the trunk was a compromise between the forehead and the wrist. The first one would be stigmatizing and could hinder enrollment and observance. The second one is subject to higher within-subject variability and higher variability between subjects than the trunk. Even though a dosimeter on the trunk underestimates UV exposure on the face (33), this underestimation is true for all participants, and the measurement is stable across participants, thus unlikely to bias our results.

## Conclusion

Over the past few years, consumers have learned about their health by using sensors in wearable devices such as smartwatches. This clinical trial is the first to quantify the impact of an accurate wearable UV dosimeter on skin cancer prevention for an elderly population. Because of the small size of our trial, our findings need to be further validated through a larger prospective trial. However, the clinical advantages observed in this pilot study indicate that using wearable UV sensors could enhance traditional UV-prevention strategies. This approach can assist patients in managing and adjusting their UV exposure habits and could also be beneficial for younger individuals who may be more receptive to innovative wearable sensor technology. Wearable UV sensors may therefore offer significant potential in substantially lowering cumulative UV exposure throughout the lives of younger patients, thereby reducing their risk of developing skin cancer in later years.

## Data availability statement

The raw data supporting the conclusions of this article will be made available by the authors, without undue reservation.

## Ethics statement

The studies involving humans were approved by Institutional Review Board of Weill-Cornell Medicine. The studies were conducted in accordance with the local legislation and institutional requirements. The participants provided their written informed consent to participate in this study.

## Author contributions

ED: Conceptualization, Formal analysis, Funding acquisition, Investigation, Methodology, Project administration, Resources, Supervision, Validation, Visualization, Writing – original draft, Writing – review & editing. PK: Conceptualization, Data curation, Formal analysis, Funding acquisition, Investigation, Methodology, Project administration, Resources, Software, Supervision, Validation, Visualization, Writing – review & editing. CD: Conceptualization, Formal analysis, Funding acquisition, Methodology, Writing – review & editing. SB: Funding acquisition, Writing – review & editing. MB: Data curation, Project administration, Writing – review & editing. KE: Formal analysis, Methodology, Supervision, Writing – review & editing. JZ: Conceptualization, Data curation, Funding acquisition, Investigation, Methodology, Project administration, Resources, Supervision, Writing – review & editing. GV: Data curation, Investigation, Project administration, Resources, Supervision, Writing – review & editing.
