# Supplementary material for: A randomized trial of a wearable UV dosimeter for skin cancer prevention
Source: Front Med (Lausanne). 2024 Mar 1;11:1259050. doi: 10.3389/fmed.2024.1259050 (PMC10940533; doi:10.3389/fmed.2024.1259050)
Supplement: Supplementary file 1 [file Data_Sheet_1.PDF]

A Randomized Trial of a Wearable UV dosimeter for Skin Cancer Prevention

SUPPLEMENTARY INFORMATION

Emmanuel LP Dumont, Ph.D.*, Peter D Kaplan, Ph.D., Catherine Do, M.D., Ph.D., Shayak Banerjee, Ph.D., Melissa Barrer, M.M.S., Khaled Ezzedine, M.D., Ph.D., Jonathan H Zippin, M.D., Ph.D., George I Varghese, M.D.

em@shade.io

Breakdown of non-melanoma skin cancers per visit

	**Control (n = 43)**	**Device (n = 49)**
**Squamous Cell Carcinoma (SCC)**		
lesions at enrollment (mean)	2 (0.05)	1 (0.02)
lesions at 3 months (mean)	1 (0.02)	2 (0.04)
lesions at 6 months (mean)	4 (0.09)	0 (0)
**Basal Cell Carcinoma (BCC)**		
lesions at enrollment (mean)	3 (0.07)	11 (0.22)
lesions at 3 months (mean)	0 (0)	2 (0.04)
lesions at 6 months (mean)	6 (0.14)	2 (0.04)

Note:

- In the device group, the 3 SCC were squamous cell carcinoma in situ (SCCIS) and were diagnosed in sun-exposed areas.
- In the control group, 6 SCC diagnosed in the control group were SCCIS and 1 was a keratoacanthoma-like SCC. 5 were diagnosed in sun-exposed skin (scalp, face, arms, lower legs) and 2 were diagnosed in non sun-exposed areas (back of the knee, thigh).
- In the device group, 10 BCC were nodular, 1 was superficial, 3 were superficial/nodular, and 1 was nodular/infiltrative. 12 were diagnosed in sun-exposed skin and 3 in non sun-exposed skin.

- In the control group, 5 BCC were nodular, 2 were mixed superficial nodular, 1 was superficial, and 1 was infiltrative. 6 of them were in sun-exposed areas and 3 in non sun-exposed areas (left breast, left calf, back).

Number of actinic keratosis per visit:

	**Control (n = 43)**	**Device (n = 49)**
**Actinic Keratoses**		
Number of lesions		
lesions at enrollment (mean)	271 (6.5)	304 (6.2)
lesions at 3 months (mean)	198 (4.5)	263 (5.2)
lesions at 6 months (mean)	183 (4.2)	194 (4.0)
